# Cost-effectiveness analysis (CEA) of IMRT plus C12 boost vs IMRT only in adenoid cystic carcinoma (ACC) of the head and neck

**DOI:** 10.1186/s13014-019-1395-9

**Published:** 2019-11-06

**Authors:** A D Jensen, Jürgen Debus

**Affiliations:** 1 Department of Radiation Oncology, University Hospital Gießen and Marburg GmbH (UKGM), Philipps-University of Marburg, Baldingerstraße, D-35032 Marburg, Germany; 2 Department of Social Policy, London School of Economics, Houghton Street, London, WC2A 2AE UK; 3 Department of Radiation Oncology, University of Heidelberg, INF 400, D-69120 Heidelberg, Germany

**Keywords:** Cost-effectiveness, CEA, IMRT, C12, Adenoid cystic carcinoma, ACC, Malignant salivary gland tumours, MSGTs

## Abstract

**Background:**

Particle therapy provides steep dose gradients to facilitate dose escalation in challenging anatomical sites which has been shown not only to improve local control but also overall survival in patients with ACC. Cost-effectiveness of intensity-modulated radiotherapy (IMRT) plus carbon ion (C12) boost vs IMRT alone was performed in order to objectivise and substantiate more widespread use of this technology in ACC.

**Methods:**

Patients with pathologically confirmed ACC received a combination regimen of IMRT plus C12 boost. Patients presenting outside C12 treatment slots received IMRT only. Clinical results were published; economic analysis on patient-level data was carried out from a healthcare purchaser’s perspective based on costs of healthcare utilization. Cost histories were generated from resource use recorded in individual patient charts and adjusted for censoring using the Lin I method. Cost-effectiveness was measured as incremental cost-effectiveness ratio (ICER). Sensitivity analysis was performed regarding potentially differing management of recurrent disease.

**Results:**

The experimental treatment increased overall costs by € 18,076 (€13,416 – €22,922) at a mean survival benefit of 0.86 years. Despite improved local control, following costs were also increased in the experimental treatment. The ICER was estimated to 26,863 €/LY. After accounting for different management of recurrent disease in the two cohorts, the ICER was calculated to 20,638 €/LY.

**Conclusion:**

The combined treatment (IMRT+C12 boost) substantially increased initial and overall treatment cost. In view of limited treatment options in ACC, costs may be acceptable though. Investigations into quality of life measures may support further decisions in the future.

## Background

Treatment with charged particles is a comparatively new and expensive radiotherapy technology. Construction and operational costs of particle facilities are estimated at more than twice the costs of standard photon therapy facilities [1–4], hence particle therapy is available in few specialised centres only. It is used to treat comparatively radioresistant tumours at complex anatomical sites [5–7] where dose escalation with photon radiotherapy is limited by normal tissue tolerance [7, 8]. In contrast to photons, charged particles such as protons or carbon ions (C12) loose most of their energy at the end of their path (Bragg peak) while depositing only little dose in the tissue beyond. Dose to adjacent critical structures is minimized and low-dose exposure to surrounding tissues is reduced.

In adenoid cystic carcinoma (ACC), dose escalation with C12 results in improved tumour control [9–12] while maintaining consistently moderate toxicity according to Japanese and German [5–7]. In a recent update, patients receiving IMRT plus C12 boost not only showed improved local control but also superior overall survival as compared to patients treated with IMRT only [13]. Due to limited availability of particle therapy and rarity of the disease [13, 14], no randomized trials have been performed to compare standard photon and carbon ion therapy. Despite approval of this treatment for ACCs in Germany following publication of initial favourable results in 2004, it is still a matter of considerable debate in the international radiotherapy community whether particle therapy is cost-effective.

While the last decade has seen a surge of interest in particle therapy, no detailed cost-effectiveness analysis based on patient-level particle data has ever been performed.

We present our economic evaluation of IMRT plus C12 boost in ACC of the head and neck using patient-level data of the two patient cohorts recently published [13].

## Methods

### Clinical data

All patients with pathologically confirmed ACC of the head and neck treated with curative intent at Heidelberg University Hospital between 1997 and 2009 were entered into the clinical analysis. Patients undergoing re-irradiation were excluded. The dataset contained information on patient baseline characteristics, initial treatment, time and incidence of locoregional or distant relapse, toxicity, last contact or death, which was previously extracted from individual patient records. Information on further treatment regarding the underlying disease was available at a basic level [13].

Within the German C12 pilot project between 1997 and 2008, C12 was clinically available in three treatment periods (20 days) per year; outside these periods, patients were treated with standard IMRT by the same team of physicians. Selection of patients into either of the cohorts was solely based on availability of C12 and hence time of initial presentation. Both cohorts are similar in their baseline characteristics and offer the unique possibility to analyse two comparable patient groups as a quasi-randomized study population. Treatment in the C12 cohort (58 patients) consisted of 30 fractions photon intensity-modulated radiotherapy (IMRT) plus 6 fractions C12 whereas the other cohort (37 patients) received 32 fractions photon IMRT. Follow-up recommendations were identical for both groups.

### Economic perspective

The analysis assesses cost-effectiveness from a health care purchaser’s perspective as represented by statutory German sickness funds. Only direct health service costs were evaluated for the duration of available follow-up. Indirect costs such as family/ caretaker leisure time, productivity losses, patient travel or hotel costs are disregarded. Costs are in € at the 2015 price level, price changes over time due to i.e. inflation were not considered.

### Costs

Cost data were derived from individual patient histories through number and category of events, resource utilization, and attributable unit costs. However, only events related to the initial tumour diagnosis were recorded. Information on supportive treatments (dietician visits, physiotherapy, pain medication) or any non-cancer related problems were not included.

Recorded resource utilization was either associated with the standard follow-up such as diagnostic procedures (CT or MRI) or associated with treatment of either local or distant tumour recurrence, therefore derived costs are limited to tumour-related health care costs.

Individual follow-up examinations were not explicitly recorded. Type of examination, time and frequency of diagnostic follow-up procedures were derived from the institution’s standard follow-up scheme [15]. Information on further treatment was available at a basic level: salvage surgery, re-irradiation and type of radiotherapy, chemotherapy or other treatments and did not include exact details or specifics on these procedures. To attribute costs, procedures were derived from standard operating procedures and expert advice. For salvage surgery, details were derived from initial tumour site and discussed with experienced head and neck as well as maxillofacial surgeons. For re-irradiation, it was assumed that patients underwent a complete second course with adequate radiation dose corresponding to approximately 30 fractions. For palliative radiotherapy, the institutional standard protocols prescribe 30 Gy in 10 fractions using standard techniques both for palliative bone and whole brain irradiation. For chemotherapy, international guidelines [16] recommend only one standard palliative regimen for recurrent or metastatic ACC consisting of a triple drug combination [17]. Patient care during chemotherapy was discussed with an experienced medical oncologist to identify i.e. appropriate number of visits, lab checks, obligatory co-medication and supportive treatment. Items therefore reflect the institution’s current clinical practice. While there are a few experimental second line chemotherapy regimens available, no consensus exists regarding their routine use on failure of first line chemotherapy. No information on second-line treatments was available for patients in this analysis hence only first line chemotherapy was modelled. Any potential second line treatment was disregarded.

### Unit cost data

Following attribution and detailed characterization of treatments or procedures to individual patient events, unit costs were extracted from official 2015 fee schedules. It was assumed that all treatments except surgical procedures were carried out on outpatient basis where costs are calculated according to uniforme reimbursement (EBM) specialist catalogues 2015 [18, 19]. University outpatient flat fees negotiated with the Association of Statutory Health Insurance Physicians are not considered. Costs for in-hospital procedures were derived from the German DRG reimbursement scheme [20–22].

C12 radiotherapy reimbursement is subject to negotiation with statutory sickness funds who kindly provided this information [23].

Drug costs for chemotherapy were obtained from AiDKlinik® search engine [24] as official pharmacy prices, potential quantity discounts for large institutions were not taken into account. All costs are reported in 2015 values (€).

#### Outcomes

While information on treatment-related acute and late toxicity was collected for clinical analysis, no information on quality of life measures is available. Primary measure of effectiveness in this analysis therefore is survival and life years gained.

#### Analysis

Cost-effectiveness results are reported as mean values with standard deviations. In order to account for censoring especially in the C12 cohort, costs were adjusted using the Kaplan-Meier sample average or Lin1 estimator proposed by Lin [25].

Both costs and effects were discounted equally to present values using recommended rates of 3.5% [26] and 3.0% [27]. Cost-effectiveness is measured as incremental cost-effectiveness ratio ($ICER=\frac{\Delta  Costs}{\Delta  Effects}$). In order to account for uncertainty, 95% confidence intervals (CI) were estimated by a non-parametric bootstrapping method (5000 samples) [28] and the Fieller method [29].

#### Sensitivity analysis

Sensitivity analysis addressed management of local tumour relapse. Re-irradiation in the C12 cohort was frequently offered as C12 at consequently higher costs, re-irradiation in the standard cohort was often carried out as standard photon radiotherapy. Two scenarios were evaluated: scenario 1 assumes re-irradiation is always carried out as standard RT, in scenario 2 always as C12.

#### Calculations and software

All calculations were performed using Microsoft® Excel® 2010 software package and Visual Basic bootstrapping macro [30]. Confidence intervals using the Fieller method were calculated with a Fieller calculation engine [31], survival probabilities with Microsoft® Excel® 2010 for cost-effectiveness analysis.

#### Ethics approval

Retrospective analysis of clinical data was approved by the institutional review board (S-141/2014 and S-492/2010) and conducted in accordance with the Declaration of Helsinki in its current version.

## Results

Mean overall survival in the IMRT+C12 group is 78.3 vs. 68.4 months in the standard treatment group [13]. At the time of evaluation, 30 patients in the standard and 26 patients in the experimental group were deceased. Sixty-two percent in the standard group and 50% in the experimental group developed locally recurrent disease (Table 1).

**Table 1:** Patient characteristics and further treatment of local or distant tumour relapse

	standard group (IMRT only)	experimental group (IMRT + C12)
number of patients	37	58
deceased	30 (81%)	26 (45%)
mean OS (months)	68.4	78.3
local relapse	23 (62%)	29 (50%)
surgery	4 (17%)	17 (59%)
re-RT	14 (61%)	13 (45%)
re-RT as C12	1 (8%)	8 (62%)
chemotherapy	1 (4%)	
distant metastases	16 (43%)	22 (38%)
chemotherapy	16 (100%)	21 (95%)
palliative RT	5 (31%)	8 (36%)

Median overall survival in the IMRT+C12 group was 102.1 months vs 73.7 months in the IMRT only group (*p* = 0.015) [13].

### Derivation of costs

Current reimbursement for C12 is a flat fee of €14,000.- [23], together with the IMRT part, the costs of the experimental treatment add to a total of € 16,877.67 vs. € 5675.27 in the standard treatment, cost categories and unit costs identified from individual patient histories are listed in Table 2.

**Table 2:** Treatment reimbursement fees, further follow-up and treatment costs given in € (2015)

category/ procedure	unit cost (€)	source
treatment		
C12 (6 sessions)	14,000.00	flat fee
IMRT 30 fractions	3877.67	EBM
IMRT+C12	17,877.67	flat fee/ EBM
IMRT 32 fractions	5675.27	EBM
follow-up		
MRI brain	124.60	EBM
MRI neck	124.60	EBM
CT chest	67.79	EBM
US abdomen	16.13	EBM
treatment of local relapse		
surgery	9725.91 - 22,789.65	DRG
re-RT standard	3983.47	EBM
re-RT C12	29,000.00	flat fee
chemotherapy (6 cycles)	7008.44	EBM
treatment of distant metastases		
chemotherapy (6 cycles)	7008.44	EBM
palliative RT	650.05	EBM

### Mean costs

Mean costs were calculated undiscounted and discounted at 3 and 3.5%. Mean difference in total costs is € 16,937 undiscounted vs. €16,035 and € 15,900 discounted at 3 and 3.5% respectively. Heavy censoring was present in the IMRT+C12 group with 55% incomplete cost observations, hence the Lin1 method was used to calculate adjusted mean differences ranging between €21,051 (3.5% discount rate) and €23,624 (undiscounted) (Table 3).

**Table 3:** Costs by cost category, mean costs and cost differences discounted, adjusted and unadjusted, given in € (2015)

	mean costs (SD) per patient						
category	standard (IMRT only) [€]	SD (IMRT only)	experimental (IMRT + C12) [€]	SD (IMRT + C12)	mean cost difference [€]	95% CI, non-parametric bootstrapping (€)	*p*-value
initial treatment	5.68	–	17.88	–	11.2	–	
diagnostic follow-up	2.66	1.47	3	1.11	332	− 219; 955	0.165
treatment of recurrence							
surgery	2.03	6.37	4.82	8.35	2.78	8; 6.039	0.002
re-RT	2.18	4.92	4.93	11.98	2.75	− 813; 6.376	0.179
chemotherapy	3.03	3.42	2.46	3.3	− 572	− 2023; 834	0.378
palliative RT	105	287	103	267	−3	− 131; 105	0.688
total complication costs	7.35	8.51	12.31	16.99	4.95	192; 10,501	0.019
mean total costs (unadjusted)							
undiscounted	15.76	9.17	33.97	17.55	18.15	12,770; 23,745	0.000
3.5% discount p.a.	14.75	7.95	32.86	14.83	18,076	13,416; 22,922	0.000
3.0% discount p.a.	14.88	8.1	32.13	15.18	17.25	12,551; 21,946	0.000
mean total costs (adjusted Lin1)							
adjusted (3 monthly intervals)	16.16		38.98		22.82		
adjusted annually	12.11		36.71		24.6		
3.5% discount p.a.	11.68		33.71		22.03		
3.0% discount p.a.	11.74		34.09		22.35		

### Incremental cost-effectiveness ratio (ICER)

Mean difference in effects is 0.86 years (undiscounted) vs. 0.83 and 0.82 years using an annual discount rate of 3 and 3.5% respectively giving rise to incremental cost-effectiveness ratios of 20,854 €/LY and 22,078€/LY; adjusted values are 26,929 €/LY (3.0% discount) and 26,863 €/LY (3.5% discount).

**Fig. 1:**
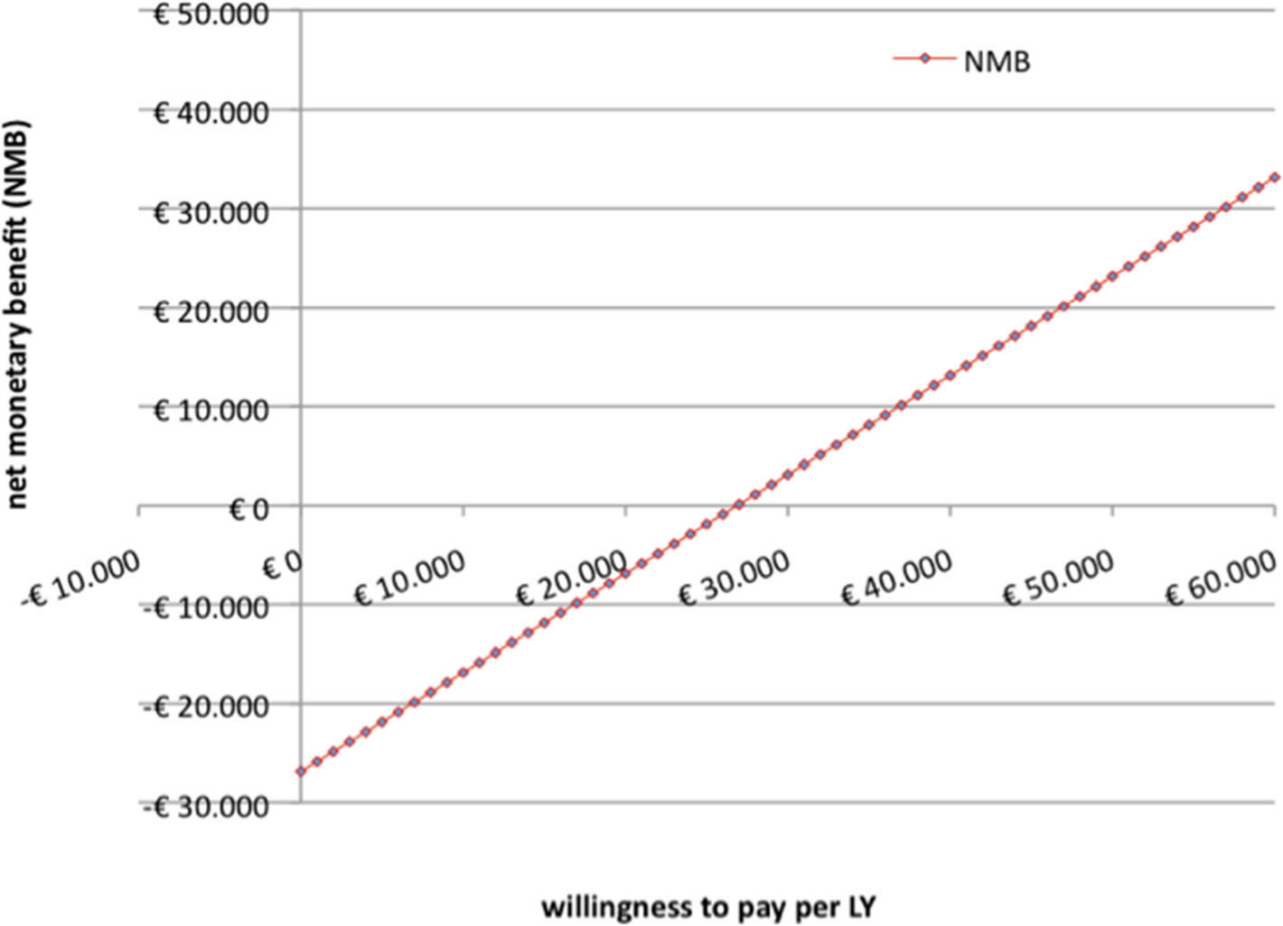
Net monetary benefit (NMB) as a function of willingness to pay per LY (adjusted, 3.5% discounting)

The net monetary benefit (NMB) as a function of the decision maker’s willingness to pay based on adjusted and discounted ICERs is shown in Fig. 1. When willingness to pay exceeds € 26.863, the NMB turns positive and the experimental treatment becomes acceptable.

### Sensitivity analysis

In the experimental treatment, re-irradiation on local relapse was more frequently offered as another course of C12 as compared to the standard treatment (Table 1). Costs for re-irradiation with C12 are substantial; hence this potential confounder was addressed. Calculations were performed for two scenarios: the first scenario assumes that all patients undergoing re-irradiation received this treatment as standard treatment (IMRT). The second scenario assumes re-irradiation always as C12. Corresponding costs were replaced in individual patient histories accordingly (Table 4).

**Table 4:** Sensititvity analysis: mean costs/ cost differences depending on management of local relapse, given in € (2015)

	IMRT	IMRT + C12	difference
mean total costs (€)			
*re-RT always as IMRT*			
mean unadjusted	15,078.-	29,636.-	14,558.-
mean adjusted (3 monthly intervals)	15,204.-	31,859.-	16,655.-
mean adjusted annually	12,066.-	30,108.-	18,042.-
mean adjusted, 3.5% discount	11,635.-	28,558.-	16,923.-
mean adjusted 3.0% discount	11,694.-	28,758.-	17,065.-
mean total costs (€)			
*re-RT always as C12*			
mean unadjusted	24,570.-	36,554.-	11,984.-
mean adjusted (3 monthly intervals)	25,058.-	41,745.-	16,687.-
mean adjusted annually	18,707.-	39,272.-	20,565.-
mean adjusted, 3.5% discount	17,810.-	36,050.-	18,240.-
mean adjusted 3.0% discount	17,931.-	36,459.-	18,528.-

ICER calculations suggests that the differing management of local relapse did indeed influence costs and hence the ICER. Both scenarios show a lower ICER with scenario one (re-irradiation as IMRT in all cases) showing the lowest value at 20,638 €/LY (Table 5).

**Table 5:** ICER depending on management of local relapse, given in € (2015)

		bootstrapped CI		Fieller CI	
	ICER (€/LY)	lower 95% (€)	upper 95% (€)	lower 95% (€)	upper 95% (€)
mean total costs: re-RT always as IMRT					
no discount	16.83	− 146.34	180.76	−20,478	5788
3.5% discount	17.43	− 147.08	174.73	−31,332	6617
3.0% discount	17.38	−144.36	207.18	−29,647	6512
*adjusted (Lin1):*					
adjusted, no discount	20.98				
adjusted, 3.5% disc.	20.64				
adjusted, 3.0% disc.	20.56				
mean total costs: re-RT always as C12					
no discount	14.64	− 122.02	124.56	−15,476	3849
3.5% discount	12.86	−104.32	139.57	−20,367	3475
3.0% discount	12,840	−102.66	148.36	−19,130	3410
*adjusted (Lin1):*					
adjusted, no discount	23.91				
adjusted, 3.5% disc.	22.24				
adjusted, 3.0% disc.	22.32				

## Discussion

With increasing number of particle projects in Europe, cost-effectiveness of this treatment technique is a matter of continuous discussion. Few institutions can offer both particle and standard photon radiotherapy techniques. Treated indications are often rare diseases, hence prospective or even randomized trials are rare. Medical technology however, has been identified as one of the major cost drivers in health care [32–34]. In view of increasing pressure on health systems and high particle facility construction and operating costs, radiation oncology can no longer avoid justifying treatments in terms of their cost-effectiveness. Reflecting these discussions, there are some modelling studies addressing CE of proton RT in prostate cancer based on Medicare data [35] and paediatric tumours based on literature reviews [36, 37]. In head and neck squamous cell carcinoma, modelling studies address appropriate patient selection [38–40] but so far have not attempted a full economic analysis. The present work is the first patient-level cost-effectiveness analysis comparing standard photon and particle radiotherapy for head and neck malignancies.

The analysis is based on two well-characterised patient cohorts treated with either standard photon RT or C12 plus photon RT at the same institution during the same time period. Selection into either of the two treatments was based on time of presentation as the experimental treatment was not continuously available. Patient baseline characteristics did not differ significantly between the two groups, and treatment preparation, and follow-up were similar [13]. Both cohorts included more than 90% T4 tumours and around 60% tumours with skull base invasion, observed benefits of carbon ion therapy on local control and overall survival in the experimental cohort may vary in patients with smaller tumours and less challenging sites. While a randomized trial setting would have been preferable in order to exclude potential confounders, due to both limited availability of the experimental treatment and rarity of the disease, the two cohorts represent a feasible approach. Authorities approved the experimental treatment as standard of care for ACC in 2005 following publication of initial experience, consequently, patients are now entitled to receive this treatment. A randomized trial to formally compare photon and particles will therefore be problematic in this setting. The underlying dataset is therefore probably the best comparison between C12 and standard RT for this disease that will be possible in Germany.

There are differences in the management of tumour recurrence between the groups though: while more patients developed local recurrences in the standard group, a higher proportion of locally recurrent patients in the experimental group underwent salvage surgery. In the standard group, percentage of patients undergoing re-irradiation for local relapse was higher than in the experimental group, however, patients in the experimental group were more likely to receive re-irradiation as C12. Observed costs reflect these findings: mean costs for surgery as well as total costs for treatment of tumour recurrence are significantly higher in the experimental group (Table 4). This is in contrast to Jäkel et al. who postulated cost savings by reducing local relapse and hence reducing the amount of surgical procedures [41]. One possible explanation is that recurrent tumours in the experimental group are still amenable to salvage surgery whereas this might not be the case in the standard group.

Mean adjusted total costs were estimated at €12,111 in the standard and €36,713 in the experimental group. As expected in the presence of 55% censoring (experimental group), accounting for incomplete cost information [25] leads to significant corrections (€11,678 and €33,706 with 3.5% discounting) (Table 3). The ICER using adjusted and discounted CE data is therefore €26,863. Though the bootstrapped results are clustered in the upper right quadrant of the CE plane, CIs are large: differences in effects (denominator) in some of the bootstrapped pairs are very small and the ICER trends to infinity. For this reason, the Fieller method [29, 31] was used to obtain CIs.

As patients in the experimental group were more likely to undergo re-irradiation with C12 and treatment costs are significantly higher than standard RT (€29,000 vs. € 3983.47) this issue was addressed in the sensitivity analysis. Two scenarios were investigated: one where re-RT is always given as IMRT and the second where re-RT is always given as C12. Both scenarios decreased mean cost differences between the two groups and consequently also the ICER (scenario 1: € 20,638; scenario 2: € 22,244, Table 5). Scenario 1 however, is problematic: data on re-irradiation of local relapse in ACC is rare and to date, only particle experience was published for this specific situation [42]. It is doubtful whether IMRT can be substituted for C12 in re-irradiation when assuming similar control rates. Potentially, the same limitations regarding dose constraints to organs at risk apply as in the primary situation. Therefore, scenario 2 seems more realistic.

The NMB as a function of ceiling ratio (Fig. 1) graphically illustrates acceptability of the treatment based on willingness to pay with an ICER of roughly € 22,000 unadjusted / € 27,000 adjusted for censoring. As no comparable CEA has yet been undertaken and attributed costs may vary in other health systems, comparison of these findings is limited. Ramaekers et al. have established a model to assess potential benefit of proton over standard RT for patients with head and neck cancer for patient triage. Assuming a reduction in toxicity but no survival benefit in their analyses, an ICER of € 60,000/QALY was found acceptable in the Netherlands [39] with a threshold of €80,000/QALY [43]. However, the authors modelled treatment consisting of particle therapy only [39] whereas the present CEA investigated costs of a combination regimen.

This CEA has some limitations regarding cost estimation. As only tumour-related events were recorded in the dataset, other healthcare costs may be underestimated. Observed toxicities showed no severe late sequelae and no major differences between the groups [13], so high toxicity-related costs are unlikely but information on non-cancer related resource utilization and costs is clearly lacking. Also, chemotherapy costs are probably underestimated as some patients may have received second or even third line chemotherapy. Lacking consensus on standard use of these treatments and specific information in the dataset, only first-line chemotherapy was considered. Standard RT costs may be overestimated as University Hospitals negotiate flat fees for outpatient treatments with responsible authorities [44], which in the case of IMRT may be below the EBM re-imbursement level [45, 46] but vary between institutions and could not been addressed in the sensitivity analysis. Likewise, follow-up costs may vary outside this institution’s standard follow-up scheme. In view of low unit costs, the effect on total costs is probably low.

## Conclusion

As opposed to some other European countries, there is no explicit ICER threshold in Germany, ultimately it is unclear whether the experimental treatment would be acceptable based on the ICER alone. Also, data presented in this cost-effectiveness evaluation are based on retrospective analysis of two similar patient cohorts, where ideally, informed decision-making would require randomized cohorts as well as further quality of life parameters. Investigations into quality of life measures in these patients may yield valuable information in order to further elucidate this issue.

## Data Availability

All resources used to derive treatment-related costs are clearly stated in the manuscript. Clinical results of respective treatments are published and therefore also accessible on an aggregate level through the published paper.

## Abbreviations

€: Euro
ACC: Adenoid cystic carcinoma
C12: Carbon ion
CEA: Cost-effectiveness analysis
CT: Computed tomography
DRG: Diagnosis-related groups
EBM: “Einheitlicher Bemessungsmaßstab” = uniforme reimbursement specialist catalogue
ICER: Incremental cost-effectiveness ratio
IMRT: Intensity-modulated radiotherapy
LY: Life-year
MRI: Magnetic resonance imaging
NMB: Net monetary benefit
QALY: Quality-adjusted life-year
RT: Radiotherapy
